# Morphine Antidependence of* Erythroxylum cuneatum* (Miq.) Kurz in Neurotransmission Processes* In Vitro*

**DOI:** 10.1155/2016/3517209

**Published:** 2016-11-16

**Authors:** Noor Azuin Suliman, Mohamad Aris Mohd Moklas, Che Norma Mat Taib, Mohd Ilham Adenan, Mohamad Taufik Hidayat Baharuldin, Rusliza Basir, Zulkhairi Amom

**Affiliations:** ^1^Department of Human Anatomy, Faculty of Medicine and Health Sciences, Universiti Putra Malaysia, 43400 Serdang, Selangor Darul Ehsan, Malaysia; ^2^Atta-ur-Rahman Institute for Natural Product Discovery, Aras 9 Bangunan FF3, UiTM Puncak Alam, Bandar Baru Puncak Alam, 42300 Selangor Darul Ehsan, Malaysia; ^3^Faculty of Health Sciences, Universiti Teknologi MARA, Kampus Puncak Alam, 42300 Puncak Alam, Selangor Darul Ehsan, Malaysia

## Abstract

Opiate abuse has been studied to cause adaptive changes observed in the presynaptic release and the mediated-synaptic plasticity proteins. The involvement of neuronal SNARE proteins reveals the role of the neurotransmitter release in expressing the opioid actions. The present study was designed to determine the effect of the alkaloid extract of* Erythroxylum cuneatum* (*E. cuneatum*) against chronic morphine and the influences of* E. cuneatum* on neurotransmission processes observed* in vitro*. The human neuroblastoma cell line, SK-N-SH, was treated with the morphine, methadone, or* E. cuneatum*. The cell lysates were collected and tested for *α*-synuclein, calmodulin, vesicle-associated membrane protein 2 (VAMP 2), and synaptotagmin 1. The extract of* E. cuneatum* was observed to upregulate the decreased expression of dependence proteins, namely, *α*-synuclein and calmodulin. The effects were comparable to methadone and control. The expressions of VAMP 2 and synaptotagmin 1 were normalised by the plant and methadone. The extract of* E. cuneatum* was postulated to treat dependence symptoms after chronic morphine and improve the soluble N-ethylmaleimide-sensitive factor activating protein receptor (SNARE) protein involved in synaptic vesicle after.

## 1. Introduction

Chronic exposure to opiates, for example, morphine, causes the progress of plasticity in the brain, expressed by dependence and addictive symptoms. Opiate dependence, withdrawal, and relapse are contributed to societal burden as was reported by Camí and Farré [1]. Introduction of opioid drugs initiate homeostatic processes explaining the natural adaptations for opioid addiction that is reflecting the induction phase of dependency [2]. The core features of addiction to opioid drugs are listing the tolerance, the withdrawal symptoms, and uncontrollable use of the drugs [3].

Biochemical trafficking among cellular compartment is facilitated by membrane carriers, usually vesicle. A number of proteins are required for the budding, target selection, and fusion of the carriers. Neurons are communicating through two main mechanisms: the secretion and reception of chemical messenger called neurotransmitter and the direct transfer through gap junctions. This study was focused on the communication via neurotransmitter [4]. Classical synaptic neurotransmitter release is facilitated by the synaptic vesicle exocytosis [5]. The classical synaptic release classical neurotransmitter is, namely, gamma aminobutyric acid (GABA), glutamate, adenosine triphosphate (ATP), acetylcholine, and glycine. Meanwhile, the monoaminergic neurotransmitters are released by exocytosis of the small dense-core vesicle from the axonal varicosities [5]. The neurotransmitters that used the core vesicle are dopamine, adrenaline, serotonin, noradrenaline, and histamine. Most neurotransmitter release is mediated by the same fundamental mechanism that involves four classes of proteins, namely, SNARE proteins, Rab-proteins, SM-proteins, and Rab-effectors [6]. The SNARE proteins and SM-proteins are responsible for catalysing the fusion reaction taking place on the presynaptic membrane, while Rab-proteins and Rab-effectors are taking role during docking and fusion reaction between the synaptic vesicle and presynaptic membrane [6].

Through proteomic analysis done by Bu et al. [7], a number of proteins including *α*-synuclein, calmodulin, and SNARE proteins were identified as being involved in morphine dependence and withdrawal and were observed in the brain. Expression of *α*-synuclein is observed to be involved in synaptic vehicle cycling and synaptic plasticity [8]. The high expression of *α*-synuclein in the brain inhibits the activity of enzymes involved in dopamine synthesis [9], thus affecting the function of dopamine transporter [10], and inhibits the release of dopamine [11], thus preventing the neurosecretion [12]. The effect of *α*-synuclein on the release of dopamine is related to the various mechanism including the secretory vesicles, trafficking to release site and Ca^2+^ dependence [12]. Supported by the study done by Martinez et al. [13], the interaction between *α*-synuclein and calmodulin in the Ca^2+^-dependent manner influences the neuroadaptation and neurotoxicity affected by the chronic morphine treatment. Calmodulin is a synaptosomal calcium-binding protein that intermediates the action of calcium, thus influencing the release and synthesis of neurotransmitter [14]. Calmodulin responded to *α*1 subunit of Ca^2+^ to the fusion machinery responsible for the release of neurotransmitter from presynaptic terminal [15].

SNARE or soluble N-ethylmaleimide-sensitive factor attachment protein receptor is a family of proteins that regulates the membrane fusion of synaptic vesicles and thus mediates the release of neurotransmitter [16]. Jahn and südhof [17] in their review had revealed a list of SNARE proteins and their intracellular localisation. Vesicle-associated membrane protein 2 (VAMP 2), synaptosome-associated protein- (SNAP-) 25, and syntaxin 1A are SNARE proteins responsible for exocytic events. The exocytic event is a release of neurotransmitter from the presynaptic membrane as the response to calcium ion (Ca^2+^) influx [18]. The complex of VAMP 2, syntaxin, and SNAP-25 was observed to cause membrane fusion, thus being responsible for membrane trafficking steps [19]. Synaptotagmin 1 is alike VAMP 2 but Ca^2+^-independent. Synaptotagmin 1 is a SNARE on the synaptic vesicles in the presynaptic terminal, acting to dock and assemble the SNARE complex. Through interaction with SNAP-25, synaptotagmin 1 facilitates the release of neurotransmitter [20].

Several pharmacotherapeutic approaches are introduced to treat opiate withdrawal such as methadone. Methadone is *μ*-opioid receptor agonist used to lessen the cravings and withdrawal symptoms in opioid addicts [21]. In the present study, chronic treatment of morphine was administrated* in vitro* in order to postulate the antidependence effect of* E. cuneatum*. The dependence property was measured through the expression of *α*-synuclein, calmodulin, VAMP 2, and synaptotagmin 1. The present study provides new insight into neurobiological and molecular changes associated with chronic morphine exposure in the cell line, which may help in developing a new pharmacotherapy derived from* E. cuneatum*.

## 2. Materials and Methods

### 2.1. Materials

Human neuroblastoma cells line SK-N-SH (ATCC® HTB­11™) were purchased from American Type Culture Collection (ATCC, USA). Minimum essential medium (MEM), fetal bovine serum (FBS), penicillin-streptomycin, Trypsin, and phosphate buffered saline (PBS) solutions were obtained from Gibco Life Technologies (Invitrogen, USA). Dimethyl sulfoxides (DMSO), retinoic acid (RA), thiazolyl blue tetrazolium bromide (MTT), and forskolin were purchased from Sigma-Aldrich (USA). Morphine sulphate pentahydrate (M-35-SU) and d,I-methadone.HCl (MET-637) were purchased from Lipomed AG (Switzerland). Isobutylmethylxanthine (IBMX) and radioimmunoprecipitation assay (RIPA) buffer and protease inhibitor were purchased from Amresco, USA. The antibodies used, *α*-synuclein, calmodulin, synaptotagmin 1, VAMP 2, anti-*β*-actin, and horseradish peroxidase (HRP) were purchased from Cell Signaling Technology (Massachusetts). WesternBright™ ECL and WesternBright Peroxide were purchased from Advansta (USA).

### 2.2. Plant Extraction

*E. cuneatum* leaves were collected from Bukit Broga, Selangor, Malaysia. The plant was sent to botanist of Universiti Putra Malaysia (UPM), Malaysia, for species verification and identification. The voucher specimen obtained is SK2100/12.

The method of the alkaloid extract was optimised by Forest Research Institute of Malaysia (FRIM). The dried leaves of* E. cuneatum* were ground into powder form and weighted. The powder leaves (750 g) were soaked in four litres of methanol for seven days until all methanolic compounds were extracted. The methanolic solution was evaporated using rotary evaporator at 45°C yielding approximately 400–500 g of methanolic extract. The methanolic extract was soaked and stirred in four litres of 10% of acetic acid glacial for overnight. Later, the mixture was filtered and fractionated in n-hexane with one to one ratio. The solution at the bottom layer of the separatory funnel was collected. The fractionation procedure was repeated for three times using the new n-hexane. Approximately 300 mL of ammonia solution with 25% of ammonium hydroxide was added into the isolated solution (bottom layer of the separatory funnel) until pH 10.00, filtered, and fractionated in chloroform with one to one ratio. The bottom layer of solvent in the separatory funnel was isolated. The isolated extract was evaporated using rotary evaporator yielding alkaloid extract. The extract was dried in the fume hood to remove the remaining chloroform, yielding approximately 0.11 g of alkaloid extract.

### 2.3. Cell Culture

The human neuroblastoma cell line SK-N-SH (ATCC HTB­11) was cultured in complete minimum essential medium containing Earle's salt and L-glutamine without sodium bicarbonate supplemented with 10% of fetal bovine serum and 1% of penicillin/streptomycin. The neuroblastoma cells were cultured as a monolayer in an incubator at 37°C in a humidified atmosphere of 5% of CO_2_. The maintaining procedure of the cells was following prescription by the manufacturer.

### 2.4. Cytotoxicity Test of* E. cuneatum* on Cell Line

The cytotoxicity assay was carried out using thiazolyl blue tetrazolium bromide (MTT) solution following the procedure prescript by the manufacturer. The SK-N-SH cells were plated into 96-well plates at the concentration of 1 × 10^5^ cells per well. The cells were incubated at 37°C with 5% of CO_2_ for 24 hrs. The cells were treated with* E. cuneatum* alkaloid extract (range from 12.5 to 400 *μ*g/mL) for 24 hrs. After 24 hrs, the cells were treated with 0.4 mg/mL of MTT solution in MEM and incubated in humidified 5% of CO_2_ at 37°C for four hrs. Consequently, the formation of formazan crystal from MTT was dissolved by adding 100 *μ*L of DMSO per well. The plate was read using microplate reader at 595 nm of absorbance [22]. The percentage of viability was counted as the average of each reading [23]. Percentage of viability was counted as the average of treated cell divided by the average of blank (cell only) multiplied by 100.

### 2.5. Morphine Induction and Treatments

The SK-N-SH cell line was seeded in 24-well plate (2 × 10^5^ cells) for 24 hrs at 37°C with 5% CO_2_ supply. The treated cells were introduced with 10 *μ*M of retinoic acid (RA) (≥98%, HPLC, Sigma) for six days to induce partial neuronal differentiation instead of massive proliferation [24]. All drugs and extract were freshly prepared by dissolving using 0.5% of dimethyl sulfoxide (DMSO) (v/v) in MEM.

The treatment was divided into two different methods, pre- and cotreatment of morphine. For pretreatment of morphine test, the cells were introduced to 50 *μ*M of morphine sulphate pentahydrate for 24 hrs and followed by 1% of DMSO, 50 *μ*M of d,I-methadone or* E. cuneatum* alkaloid extract for another 24 hrs. As for cotreatment of morphine, the cells were treated with 50 *μ*M of morphine sulphate pentahydrate and 50 *μ*M of d,I-methadone·HCl or* E. cuneatum* alkaloid extract together for 24 hrs of incubation. As for negative control, the cells were treated with 0.5% of DMSO in MEM.

### 2.6. Expression of Endocytic Markers

The cells were washed with ice-cold PBS before being treated with 50 *μ*L of cold RIPA buffer and protease inhibitor (1 : 1000) for 15 mins on ice. The cells were scraped and collected in the ice-cold microcentrifuge tubes before being centrifuged at 15,000 g for 15 mins at 4°C. The supernatants were collected in new cold microcentrifuge tubes and stored at −80°C.

For gel preparation, 4% of stacking gel and 10% of resolving gel were used. Twenty *μ*L of the samples containing 25 *μ*g of total protein was added to 20 *μ*L of laemmli buffer and heated at 95°C for five mins. The samples were centrifuged at 1,000 rpm for one min before being loaded into the gel. The electrophoresis procedure was run at 100 V for 60 mins before being increased until 150 V for another 30 mins. Then, the gel was transferred to polyvinylidene difluoride (PVDF) membrane using wet transfer method for one hr. The membrane was incubated with 5% of skimmed milk for two hrs at 4°C. Then, the membrane was incubated with anti-*α*-synuclein (1 : 2500), anti-calmodulin (1 : 2500), anti-synaptotagmin 1 (1 : 1000), and VAMP 2 (1 : 1000) for overnight in 4°C. Then, the membrane was washed thrice with TBST 20 for five mins each time and incubated with secondary antibody HRP (1 : 5000) for two hrs at 4°C. The membrane was washed thrice with TBST for 10 mins. The membrane was coated using chemiluminescent HRP Substrate, 1 : 1 of WesternBright ECL, and WesternBright Peroxide. The membrane was viewed using Gel Documentation and the image of the protein of interest was obtained.

The membrane was incubated with stripping buffer for 5 mins at room temperature. The buffer was replaced with the fresh stripping buffer for another 5 mins. The membrane was washed with TBST 20 thrice, 15 mins for each wash. Then, the membrane was blocked with 5% of skimmed milk for two hrs at 4°C before being incubated with the *β*-actin antibody (1 : 10000) for one hr at 4°C. The membrane was washed with TBST 20 thrice, 10 mins each before being incubated with HRP antibody for one hr at 4°C. The membrane was washed and viewed using chemiluminescent HRP Substrate.

The data were normalised by dividing the mean intensity of the protein of interest with the housekeeping protein (*β*-actin) for each reading. The protein levels were presented as percentage changes compared with control treated cell, designated as 100%. One-way analysis of variance (ANOVA) was performed with IBM SPSS Statistic 21 software followed by a post hoc Tukey's multiple comparison tests where applicable for intergroup comparison, with *p* < 0.05 being considered as a significant difference.

### 2.7. Concentration of Intracellular Ca^2+^  [Ca^2+^]_i_

Concentration of intracellular Ca^2+^[Ca^2+^]_i_ was studied using the 5 × 10^5^ cells density that seeded in 96-well black plate with clear bottom plates. The cell was treated with 10 *μ*M of RA for six days, followed by chronic pre- and cotreatments of morphine. The pretreatment of morphine was given for 24 hrs followed by chronic methadone and* E. cuneatum*. The chronic cotreatment of morphine was given together with methadone and* E. cuneatum*. Later, the cells were incubated with 50 *μ*L of calcium reagent (Fluo-4 Direct™, Starter pack, F10471, Invitrogen) with 50 *μ*L of remaining media in the well for five hrs at 37°C and 5% CO_2_. The plate was read using fluorescent microplate reader (Infinite® M200, Tecan Trading AG, Switzerland) for excitation at 494 nm and emission at 516 nm. The data obtained was in the fluorescence arbitrary unit (AU). Since the procedure given by the manufacturer was not measuring the baseline fluorescence, the five hrs data was converted to the percentage. All data of control, morphine, methadone, and* E. cuneatum* were divided with control and multiplied by 100, yielding the percentage of fluorescence over control. The data were analysed using IBM SPSS Statistic for one-way ANOVA and Tukey's multiple comparison tests.

## 3. Result

### 3.1. Cytotoxicity Test of* E. cuneatum* on Cell Line

**Figure 1:**
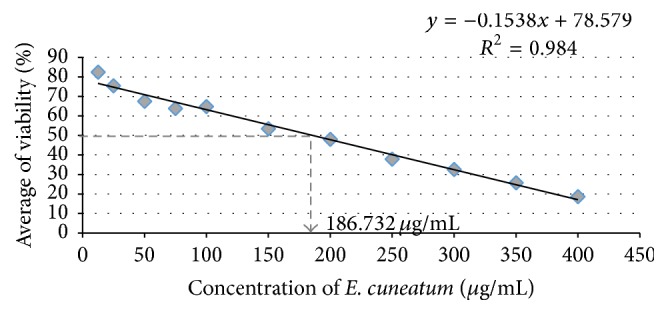
Cytotoxicity effect of the alkaloid extract of* E. cuneatum* on the neuroblastoma cell line.

The cell viability was studied using MTT assay. The dose response was plotted by the percentage of cell viability against the concentration of the* E. cuneatum* (Figure 1). The IC_50_ value of the alkaloid extract of* E. cuneatum* was approximately 186.7 *μ*g/mL.

### 3.2. Expression of Endocytic Marker

**Figure 2:**
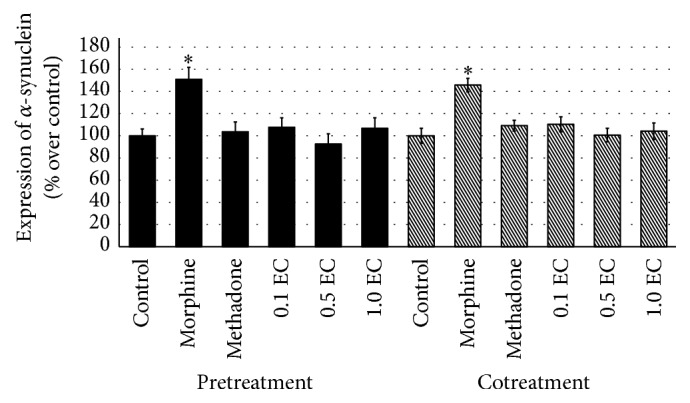
Expression of *α*-synuclein for pre- and cotreatment of morphine. The bar chart shows the percentage of altered *α*-synuclein over control for pre- and cotreatments of morphine. Increased level of *α*-synuclein in the morphine-treated cell, significant to control (^*∗*^ *p* < 0.05), was downregulated by methadone and all doses of* E. cuneatum*. The effects of methadone and* E. cuneatum* were significant to morphine treatment (*p* < 0.05), while comparable to the control. Three independent experiments were carried out and the data represent the mean percentage of [Ca^2+^]_i_ over control ± SEM. Statistical analysis was carried out using Tukey's multiple comparison tests. ^*∗*^ *p* < 0.05 versus control. (EC:* E. cuneatum;* 0.1 = 0.1 *μ*g/mL; 0.5 = 0.5 *μ*g/mL; 1.0 = 1.0 *μ*g/mL.)

**Figure 3:**
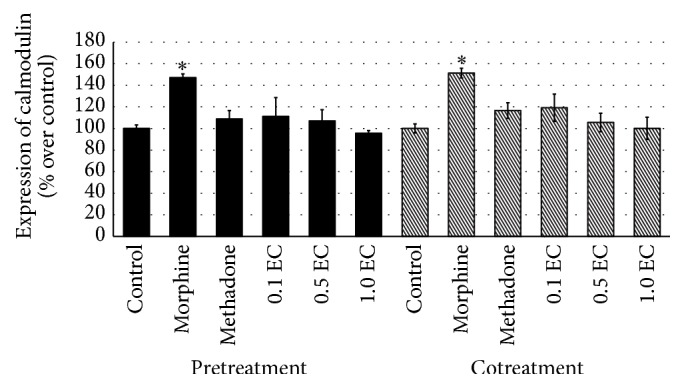
Expression of calmodulin for pre- and cotreatments of morphine. The bar chart shows the percentage of affected expression of calmodulin over control for pre- and cotreatments of morphine. The upregulation of calmodulin prior to morphine treatment was significant to control (^*∗*^ *p* < 0.05). The stimulated expression of calmodulin was observed to be normalised by methadone and* E. cuneatum*. The downregulated level of calmodulin influenced by methadone and* E. cuneatum* was significant to morphine (*p* < 0.05), while it was comparable to control. Three independent experiments were carried out and the data represent the mean percentage of [Ca^2+^]_i_ over control ± SEM. Statistical analysis was carried out using Tukey's multiple comparison tests. ^*∗*^ *p* < 0.05 versus control. (EC:* E. cuneatum;* 0.1 = 0.1 *μ*g/mL; 0.5 = 0.5 *μ*g/mL; 1.0 = 1.0 *μ*g/mL.)

**Figure 4:**
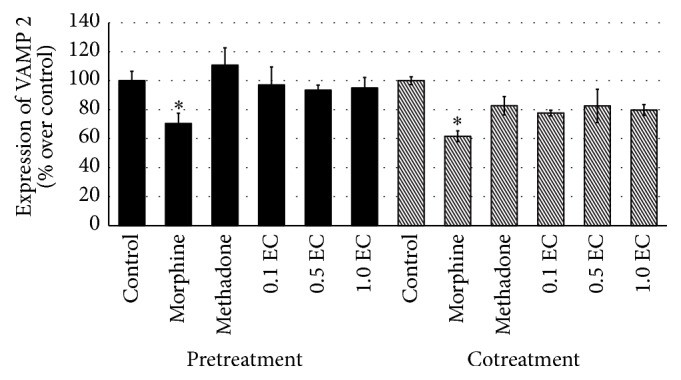
Expression of VAMP 2 observed in pre- and cotreatments of morphine. The bar chart shows the expression of VAMP 2 as expressed in percentage over control for pre- and cotreatments of morphine. The cell treated with morphine showed downregulation of VAMP 2 significant to control (^*∗*^ *p* < 0.05). The expression of VAMP 2 was elevated by the methadone and* E. cuneatum* observed in pre- and cotreatments of morphine. The upregulation of VAMP 2 by those compounds was significant as compared to morphine (*p* < 0.05) in pretreatment of morphine while comparable to control. As for cotreatment of morphine, the suppressed level of VAMP 2 prior to morphine was insignificant to the upregulation of the protein by the methadone and* E. cuneatum*. Three independent experiments were carried out and the data represent the mean percentage of [Ca^2+^]_i_ over control ± SEM. Statistical analysis was carried out using Tukey's multiple comparison tests. ^*∗*^ *p* < 0.05 versus control. (EC:* E. cuneatum;* 0.1 = 0.1 *μ*g/mL; 0.5 = 0.5 *μ*g/mL; 1.0 = 1.0 *μ*g/mL.)

**Figure 5:**
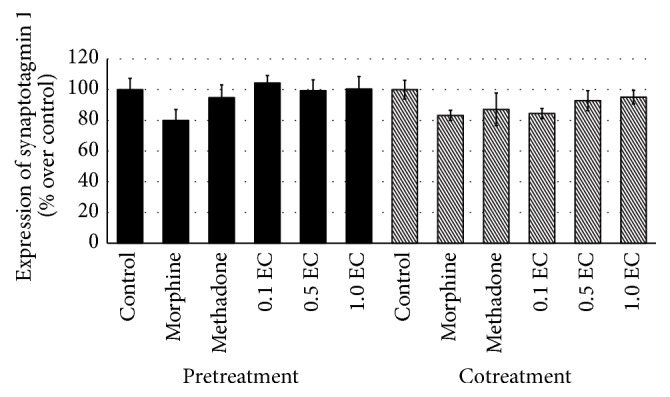
Expression of synaptotagmin 1 for pre- and cotreatments of morphine. The bar chart shows the expression of synaptotagmin 1 presented by the percentage changes over control for pre- and cotreatments of morphine. The expression of synaptotagmin 1 was observed to be downregulated prior to the treatment of morphine alone, insignificantly different to control, methadone, and* E. cuneatum*. The treatment of methadone and* E. cuneatum* was normalising the inhibited expression of synaptotagmin 1. Seven independent experiments were carried out and the data represent the mean percentage of [Ca^2+^]_i_ over control ± SEM. Statistical analysis was carried out using Tukey's multiple comparison tests. (EC:* E. cuneatum;* 0.1 = 0.1 *μ*g/mL; 0.5 = 0.5 *μ*g/mL; 1.0 = 1.0 *μ*g/mL.)

Throughout this study, three different doses of* E. cuneatum* were used, 0.1, 0.5, and 1.0 *μ*g/mL. In observing the effect of* E. cuneatum* on morphine dependence, pretreatment of morphine was introduced. The pretreatment of 50 *μ*M of morphine was suggested to exhibit withdrawal property observed in the human neuroblastoma cell line [22]. From the study, four different markers for expressing chronic morphine adaptation were used, namely, *α*-synuclein, calmodulin, VAMP 2, and synaptotagmin 1. The cells that treated with chronic morphine were expressed and significantly altered the expression of all markers, causing upregulation of *α*-synuclein (Figure 2) and calmodulin (Figure 3) while reducing the expression of VAMP 2 (Figure 4) and synaptotagmin 1 (Figure 5) significantly as compared to control (excluding synaptotagmin 1). The effected proteins level was observed to be normalised by the administration of 50 *μ*M of methadone and* E. cuneatum.* The* E. cuneatum* were observed to imitate the effects of methadone observed on all four proteins. The methadone and* E. cuneatum* downregulated the expressions of *α*-synuclein and calmodulin while upregulated the VAMP 2 and synaptotagmin 1 levels, nonsignificantly as compared to control. The doses of* E. cuneatum* used showed no significant difference as all doses gave the same effects.

Cotreatment of morphine with methadone or* E. cuneatum* was introduced to determine the pharmacological effect of those compounds, either antagonism or synergism. The treatment of morphine alone was expressed with significant high contents of *α*-synuclein (Figure 2) and calmodulin (Figure 3), with low expressions of VAMP 2 (Figure 4) and synaptotagmin 1 (Figure 5), as compared to control (excluding synaptotagmin 1). The cotreatment of morphine with methadone or* E. cuneatum* was demonstrated to significantly maintain the expressions of *α*-synuclein, calmodulin, VAMP 2, and synaptotagmin 1 as compared to morphine alone. The cells treated with morphine and methadone or* E. cuneatum* were comparable to control.

### 3.3. Intracellular Ca^2+^  [Ca^2+^]_i_

**Figure 6:**
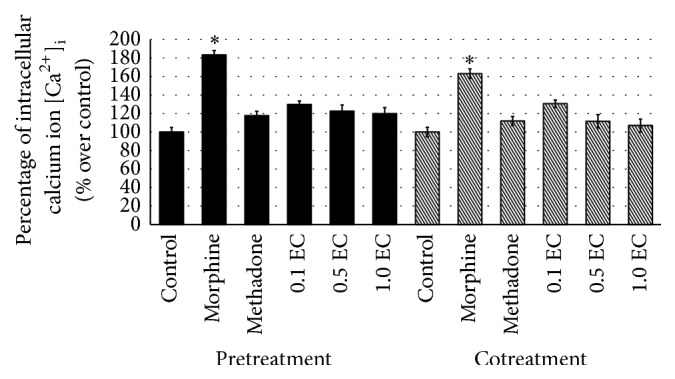
The concentration of intracellular calcium ion [Ca^2+^]_i_ for pre- and cotreatments of morphine. The bar chart shows the percentage of [Ca^2+^]_i_ over control. The [Ca^2+^]_i_ for morphine-treated cell was observed to be stimulated significantly as compared to control (^*∗*^ *p* < 0.05). The increased level of [Ca^2+^]_i_ of morphine was downregulated by the treatments of methadone and* E. cuneatum*. The treatments of methadone and* E. cuneatum* were demonstrated to downregulate the significantly increased level of [Ca^2+^]_i_ induced by morphine. There were no significant differences between the control, methadone, and* E. cuneatum*. Three independent experiments were carried out and the data represent the mean percentage of [Ca^2+^]_i_ over control ± SEM. Statistical analysis was carried out using Tukey's multiple comparison tests. ^*∗*^ *p* < 0.05 versus control. (EC:* E. cuneatum;* 0.1 = 0.1 *μ*g/mL; 0.5 = 0.5 *μ*g/mL; 1.0 = 1.0 *μ*g/mL.)

The present study has studied the effect of chronic morphine on Ca^2+^ contents. The treatment of morphine alone was observed to increase the concentration of intracellular Ca^2+^[Ca^2+^]_i_ significantly as compared to control (Figure 6). The stimulated expression of [Ca^2+^]_i_ was observed to be normalised by the treatments of methadone and* E. cuneatum*. All doses of* E. cuneatum* used were observed to nonsignificantly downregulate the level of [Ca^2+^]_i_ as compared to methadone and control. As for cotreatment of morphine, the treatment of morphine alone had increased the level of [Ca^2+^]_i_ significantly as compared to control (Figure 6). The effect of influenced [Ca^2+^]_i_ was nonsignificantly different as compared to pretreatment of morphine and even there was visible diverse. Meanwhile, the cotreated morphine with methadone was observed to maintain the level of [Ca^2+^]_i_. Interestingly, all doses of* E. cuneatum* were demonstrated to imitate the effect of methadone by sustaining the level of [Ca^2+^]_i_ as the level of control.

## 4. Discussion

The present study demonstrated that the alkaloid extract of* E. cuneatum* induced cell death at 186.7 *μ*g/mL which presented by colour changes from yellow MTT reduced to purple formazan in the mitochondria of living cells. Reduction occurs upon activation of the mitochondrial reductase and subsequently directly demonstrates the viability of the cells. By comparing the amount of purple formazan produced by* E. cuneatum*-treated cells with untreated cells, the efficiency of the* E. cuneatum* in contributing cell death can be presumed. Thus, the following receptor studies were run using safe dose at 0.1 and 1 *μ*g/mL. Expression of cAMP plays an important role in reflecting chronic morphine treatment [25]. Considering the unaffected level of cAMP, we assumed that the treatments of methadone and* E. cuneatum* did not influence the expression of proteins in the present study.

Throughout the study, human neuroblastoma cell line, SK-N-SH, was used. The main reason of using SK-N-SH is due to high expression of *μ*-opioid receptor in the cell line [26]. Human neuroblastoma cell line, SK-N-SH, was reported to contain 5 : 1 ratio of *μ*-opioid and *δ*-opioid receptor [27]. The *μ*-*δ* heteromer leads to the prominent receptor pharmacology in which the low *δ*-receptor ligands potentiate the activities of *μ*-opioid receptors. The *μ*-*δ* heteromer is also crucial for expressing the morphine-tolerance [28]. As for confirmation, receptor affinity was done to observe the responsible receptor for morphine, methadone, and* E. cuneatum*. Unpublished data showed the involvement of *μ*-opioid receptor in expressing the effects of those compounds.

Before conducting the research, the effect of methadone and* E. cuneatum* on the cell line was determined (unpublished data). The cell was treated with the same dose of methadone and* E. cuneatum*. The expression of cAMP was determined using kit. The cAMP level of the treated cells showed no difference as compared to control (5% of DMSO in complete MEM) (*p* > 0.05). Thus, it is postulated that methadone and* E. cuneatum* did not affect the healthy cells.

The roles of *α*-synuclein including in synaptic vehicle cycling and synaptic plasticity [8] are implicated in neurodegenerative diseases, for example, Parkinson's disease [29]. The neuronal protein *α*-synuclein is an important regulator of dopamine function, found in the presynaptic terminals of the neuron [30]. The high expression of *α*-synuclein in the brain inhibits the activity of enzymes involved in dopamine synthesis [9], thus affecting the function of dopamine transporter [10] and inhibiting the release of dopamine [11], thus preventing the neurosecretion [12]. The alteration in dopaminergic system in the brain stimulated by *α*-synuclein contributes to an exhibition of dependence and abuse of drug and alcohol [12, 31]. Elevated expression of *α*-synuclein was suggested to downregulate the activity of dopaminergic. Withdrawal activity [12] and morphine dependency [32] due to chronic morphine treatment were showed by the accumulation of this protein in the brain.

In the present study, cells treated with chronic 50 *μ*M of morphine showed the elevated level of *α*-synuclein expression. The upregulation of the protein expression was observed in the dopaminergic terminal, thus suggesting the role of dopamine in morphine addiction [33]. Downregulation of dopaminergic neurotransmission is related to opioid withdrawal [34] influenced by the accumulation of *α*-synuclein in the axon terminals. Surprisingly, the expression of *α*-synuclein was downregulated in the cells treated with the methadone and alkaloid extract of* E. cuneatum* interventions prior to pretreatment of morphine. All doses of* E. cuneatum* were mimicking the effect of methadone in treating chronic morphine, as methadone is well-known therapy for managing the chronic morphine [7]. The cotreatment of* E. cuneatum* and morphine was observed to downregulate the expression of *α*-synuclein reflecting the cotreatment of methadone and morphine. The observations were significant as compared to morphine treatment alone. The findings suggest that the treatments of morphine, methadone, and* E. cuneatum* did react on the same receptor. The* E. cuneatum* was found to diminish the dependency property of morphine which is insignificantly different to methadone observed in both pre- and cotreatments of morphine. All doses of* E. cuneatum* exhibited the decreased level of *α*-synuclein suggesting the antidependency property against morphine. It is postulated the involvement of* E. cuneatum* on the dopaminergic signaling pathway affects the release of dopamine from the synaptic terminal.

The effect of *α*-synuclein on the release of dopamine is related to the various mechanisms including the secretory vesicles, trafficking to release site, and Ca^2+^ dependence [12]. The interaction between *α*-synuclein and calmodulin in the Ca^2+^-dependent manner influences the neuroadaptation and neurotoxicity affected by the chronic morphine treatment [13]. Calmodulin is a synaptosomal calcium-binding protein that intermediates the action of calcium, thus influencing the release and synthesis of neurotransmitter [14]. Calmodulin responded to *α*1 subunit of Ca^2+^ to the fusion machinery responsible for the release of neurotransmitter from presynaptic terminal. The bound of calmodulin at two different subunits of the Ca^2+^ channel allows the calmodulin to detect the concentration of Ca^2+^ in the presynaptic terminal [15]. Calmodulin and Ca^2+^ are responsible for a number of the intracellular roles including neurotransmitter biosynthesis, especially in response to opioid dependence and tolerance [35]. The processes mediated by Ca^2+^ via calmodulin are responsible for the development of morphine dependence observed through the high level of calmodulin expression in the brain [36].

The present study demonstrated the upregulation of calmodulin upon the treatment of morphine alone. The finding was responding to the report of Bu et al. [7] suggesting the dependence symptom on the increased level of calmodulin in chronic morphine-treated cells. Chronic morphine induced cell was treated with methadone and* E. cuneatum*. The treatment of 50 *μ*M of methadone was studied to downregulate the expression of calmodulin as it was suggesting the antidependence effect of methadone. Interestingly, all doses of* E. cuneatum* expressed the decreased level of calmodulin, imitating the effect of methadone. The* E. cuneatum* was suggested to share the same property with methadone in treating chronic morphine as antidependence compound. In cotreatment of morphine, morphine alone was observed to elevate the expression of calmodulin, nonsignificantly as compared to pretreatment groups. The increased level of calmodulin was suggesting to express the morphine dependence property as claimed by Nehmad et al. [36]. As methadone and* E. cuneatum* reacted on the same receptor (unpublished data), the cotreatments of those compounds were observed to diminish the expression of calmodulin. The treatment of methadone had decreased the stimulated level of calmodulin to the normal level as it was known to be used to treat the effects of chronic morphine [7].

**Figure 7:**
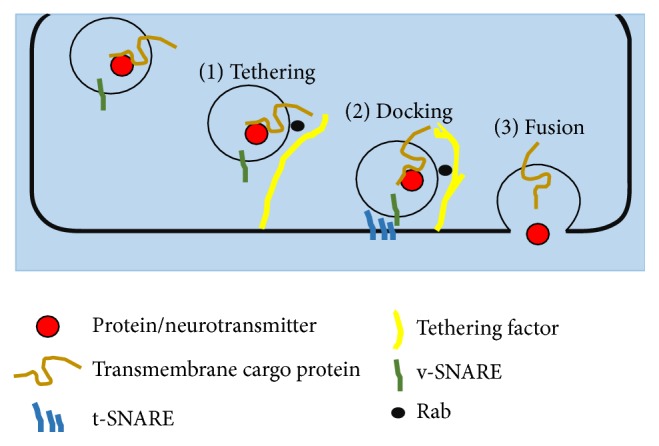
Vesicle trafficking for neurotransmission from the fusion machinery in the presynaptic terminal. The synaptic vesicle containing neurotransmitter consists of a number of vesicular SNARE (v-SNARE) such as VAMP 2, synaptotagmin 1, synapsin, calmodulin kinase II (CaMKII), and myosin-V. The activity of releasing neurotransmitter occurs in the active zone where the synaptic vesicles are accumulated and high concentration of Ca^2+^ is compulsory. During tethering phase, the vesicle moves to acceptor compartment and tethers through the organisation of a GTP-bound Rab and the tethering factor. The vesicle docks or immobilises the plasma membrane through the v-SNARE and t-SNARE complex assembling into a four-helix bundle. The SNARE complex leads the fusion of the vesicle and releases of protein or neurotransmitter which required a higher concentration of Ca^2+^. The exocytosis and endocytosis processes are taking place where the neurotransmitters are released to the synaptic gap and the vesicle is recycled (review by [43]).

VAMP 2 or synaptobrevin is also v-SNARE. Compared to synaptotagmin 1, VAMP2 is a Ca^2+^-dependent that bound to calmodulin that mediates the acidic phospholipid-binding activity of VAMP. In the same time, the complex of VAMP 2 and calmodulin prevents the formation of VAMP-SNARE complex, thus decreasing the exocytosis frequency [37]. VAMP 2 was studied to form ternary complexes with SNAP-25 and syntaxin (Figure 7) [38]. VAMP 2 is suggested to form a core complex together with t-SNARE proteins known as syntaxin and SNAP-25 [39], which contributed to the membrane fusion [40]. After fusion of SNAREs, VAMP 2 is detached from the complex, retrieved by the endocytosis, and recycled for another round of membrane fusion [41]. VAMP 2 was observed to be activated by Ca^2+^-calmodulin by extracting the juxtamembrane region from the liposome membrane [37].

The present study showed the treatment of morphine alone has decreased the expression of VAMP 2. The finding suggests that chronic morphine causes the low formation of trans-SNARE as the low number of VAMP 2 as v-SNARE to bind to syntaxin and SNAP-25. Thus, the treatment of morphine has downregulated the exocytosis of related neurotransmitter [38]. As expected, the treatment of methadone in pre- and cotreatments of morphine was observed to upregulate the inhibited VAMP 2 expression as it was supported by Mattick et al. [21]. Surprisingly, all doses of* E. cuneatum* were observed to imitate the effect of methadone in pre- and cotreatments of morphine. The* E. cuneatum* was demonstrated to increase the expression of VAMP 2 against morphine. The* E. cuneatum* was suggested to elevate the formation of SNARE complex, thus increasing exocytosis process of certain neurotransmitter. The effects of methadone and* E. cuneatum* in managing the expression of VAMP 2 and subsequently the complex of SNARE were postulated to treat the dependency, tolerance, and withdrawal of morphine [7, 21].

Like VAMP 2, synaptotagmin 1 protein is also a vesicular SNARE (v-SNARE) on the synaptic vesicles in the presynaptic terminal, responsible for docking and SNARE complex assembly (Figure 7). It interacts directly with SNAP-25 on the presynaptic membrane and thus mediates the release of neurotransmitter [20]. The influx of Ca^2+^ allowed the synaptotagmin 1 to penetrate the membrane and destabilise it and allowed the fusion to happen. Jahn et al. [6] had proposed the role of synaptotagmin 1 in promoting the vesicle fusion when the trans-SNARE complex is blocked by accessory proteins. In addition, chronic treatment of morphine was also observed to alter the synaptotagmin 1 gene, affecting the neuronal and behavioural plasticity in the mesolimbic reward system region [42].

The present study demonstrated the insignificant difference of synaptotagmin 1 expression between the morphine, methadone, and* E. cuneatum*. Despite the insignificant effects, there was visible difference between the compounds. The treatment of morphine alone was observed to suppress the expression of synaptotagmin 1 as compared to control. The low content of synaptotagmin 1 was postulated to impair the vesicle trafficking and the release of neurotransmitter. The treatments of methadone and* E. cuneatum* were studied to regulate the expression of synaptotagmin 1 by increasing the level of the protein. Thus, the vesicle fusion mechanism was restored and, subsequently, neurotransmitter was released. In cotreatment of morphine, the cotreated cell with methadone and* E. cuneatum* exhibited the similar pattern with the pretreated morphine. The effect of methadone was reflecting the outcome of the treatment of* E. cuneatum*. Thus, it was postulated that morphine, methadone, and* E. cuneatum* were reacting on the same receptor and same mechanism.

Ca^2+^ plays an important role in the action of opioids, studied tremendously in molecular mechanisms of opioid dependence, tolerance, and abstinence syndrome. The administration of opiates inhibits the depolarisation-induced influx of Ca^2+^, thus reducing the release of neurotransmitter [42]. Opiates action in inhibiting the release of transmitter from nerve terminal was reversed through the high influx of Ca^2+^ [44] mediated by the changes in Ca^2+^ uptake and binding [42]. Chronic exposure to opiates, for example, morphine, was observed to stimulate the level of Ca^2+^, thus diminishing the inhibitory effects on neurotransmitter release resulting the tolerance effect [45]. Synaptic vesicle fusion required a higher concentration of Ca^2+^ than resting stage of the neuron for endocytosis and neurotransmission to take place [46].

Findings from the present study showed the increased level of [Ca^2+^]_i_ of the morphine-treated cell, observed in pre- and cotreatment groups. The treatment of morphine alone was significantly different as compared to control supporting the report by Ansari et al. [47] and Bongianni et al. [45] suggesting the withdrawal and tolerance properties. The effect of chronic treatment of morphine was treated with methadone and* E. cuneatum*. Treatment of 50 *μ*M of methadone was demonstrated to significantly normalise the level of [Ca^2+^]_i_ upon the pretreated morphine. The downregulated [Ca^2+^]_i_ by all doses of* E. cuneatum* were reflecting the effect of methadone. Three doses of* E. cuneatum* used showed decreased level of [Ca^2+^]_i_ postulating the antidependence property of the plant. As for cotreatment of morphine, the methadone and* E. cuneatum* were studied to react on the same receptor with morphine. It was expressed by the downregulation of [Ca^2+^]_i_ on the cotreatments of morphine with methadone and* E. cuneatum*.

Through the study, chronic treatment of 50 *μ*M of morphine exhibits the dependence property expressed by a high content of *α*-synuclein, calmodulin, and [Ca^2+^]_i_ and also a low level of VAMP 2 and synaptotagmin 1. Chronic morphine was observed to inhibit neurotransmitter release through inhibition of fusion machinery. Methadone, an agonist drug against morphine is well-known to be used to manage morphine dependence, tolerance, and withdrawal. In the present study, 50 *μ*M of methadone was managed to normalise the altered proteins. The treatment of methadone was expressed the antidependence property while stimulating the vesicle fusion to the terminal membrane and thus enhanced the release of neurotransmitter. Interestingly, all doses of* E. cuneatum* were observed to reflect the effects of methadone in all proteins.* E. cuneatum* did diminish the upregulated *α*-synuclein and calmodulin, thus suggesting the antidependence property of the plant. The treatment of* E. cuneatum* also did elevate the suppressed level of VAMP 2 and synaptotagmin, while decreasing [Ca^2+^]_i_, suggesting the effect of the plant in improving vesicle trafficking and release of neurotransmitter

## 5. Conclusion

Chronic treatment of morphine was proved to cause dependence symptoms observed in the cell line. Morphine was observed to increase the level of *α*-synuclein, calmodulin, and [Ca^2+^]_i_, while decreasing the content of VAMP 2 and synaptotagmin 1. The treatments of methadone and* E. cuneatum* were surprisingly managing the adverse effect of chronic morphine by diminishing the level of *α*-synuclein, calmodulin, and [Ca^2+^]_i_, while increasing the VAMP 2 and synaptotagmin 1. All doses of* E. cuneatum* used were demonstrated to reflect the effects of methadone on the proteins.* E. cuneatum* was postulated to share the same properties of methadone. The plant was suggested to have antidependence property against morphine via improving the vesicle trafficking and release of neurotransmitter.
